# Atypical Spitz Tumor Versus Dermatofibroma: Two Case Reports Highlighting a Diagnostic Challenge

**DOI:** 10.7759/cureus.113021

**Published:** 2026-07-20

**Authors:** Zakia Douhi, Zineb Aqasbi Ouahi, Layla Tahiri Elousrouti, Laila Chbani, Fatima Zahra Mernissi

**Affiliations:** 1 Dermatology, Human Pathology, Biomedicine and Environment Laboratory, Faculty of Medicine, Pharmacy and Dentistry, Hassan II University Hospital, Sidi Mohammed Ben Abdellah University, Fez, MAR; 2 Dermatology, Hassan II University Hospital, Fez, MAR; 3 Pathology, Hassan II University Hospital, Fez, MAR

**Keywords:** atypical spitz tumor, dermatofibroma, dermoscopy, differential diagnosis, reflectance confocal microscopy, spitzoid neoplasms

## Abstract

Spitzoid tumors comprise a heterogeneous spectrum of melanocytic neoplasms, ranging from benign Spitz nevi to malignant Spitzoid melanoma, with atypical Spitz tumor (AST) representing an intermediate entity of uncertain biological potential. AST is a rare melanocytic lesion that may clinically mimic non-melanocytic tumors, particularly dermatofibroma, making accurate diagnosis challenging. It predominantly affects children and young adults and has a predilection for the lower extremities.

We describe two cases of young female patients presenting with clinically similar erythematous-pigmented plaques on the thigh. Despite comparable clinical appearances, dermoscopic evaluation revealed distinct patterns that led to different diagnoses: a spitzoid melanocytic proliferation (confirmed as AST on histology) and a dermatofibroma, with markedly divergent biological behaviors and prognostic implications.

In spitzoid lesions, the presence of dotted vessels, shiny white streaks, inverse pigment network, and/or brown globules should raise suspicion of a melanocytic spitzoid proliferation. In contrast, the dermatofibroma exhibited a peripheral pigmented network, a central scar-like white area, and brown ringed globules, consistent with the typical dermoscopic pattern of this benign fibrohistiocytic tumor.

These observations underscore the value of dermoscopy as a diagnostic triage tool in the assessment of spitzoid lesions. However, definitive diagnosis still relies on histopathological evaluation.

## Introduction

Atypical Spitz tumors (ASTs), also referred to as Spitz melanocytomas in the current WHO Classification of Skin Tumours (5th edition, 2022) [1], are spitzoid melanocytic lesions representing an intermediate category between benign Spitz nevi and Spitz melanoma [2]. Their diagnosis remains particularly challenging due to significant histopathological overlap with both entities. In addition, ASTs may clinically mimic a variety of benign and malignant lesions. Notably, ASTs can be clinically indistinguishable from dermatofibromas, as both can present as erythematous to pigmented papules or plaques on the extremities of young adults.

In this diagnostically challenging setting, dermoscopy plays a pivotal role, not in establishing a definitive diagnosis of AST but in identifying features suggestive of a spitzoid melanocytic proliferation. Reflectance confocal microscopy (RCM) can provide additional in vivo morphological information, helping to refine diagnostic suspicion and guide the decision to perform surgical excision for definitive histopathological assessment [3,4]. However, the distinction between AST and benign mimickers such as dermatofibroma remains challenging in daily clinical practice, and reports illustrating the contribution of multimodal imaging in this specific differential diagnosis are limited.

In this report, we describe two cases of young female patients presenting with clinically similar thigh lesions. Despite the clinical similarity of the lesions, dermoscopic evaluation revealed distinct patterns that led to different diagnoses: a spitzoid melanocytic proliferation (confirmed as AST on histology) and a dermatofibroma, with markedly divergent biological behaviors and prognostic implications. Through this direct comparison, we aim to emphasize the diagnostic pitfalls of clinically similar lesions and provide practical insights that may assist clinicians in distinguishing AST from benign fibrohistiocytic mimickers.

## Case presentation

Case 1

**Figure 1:**
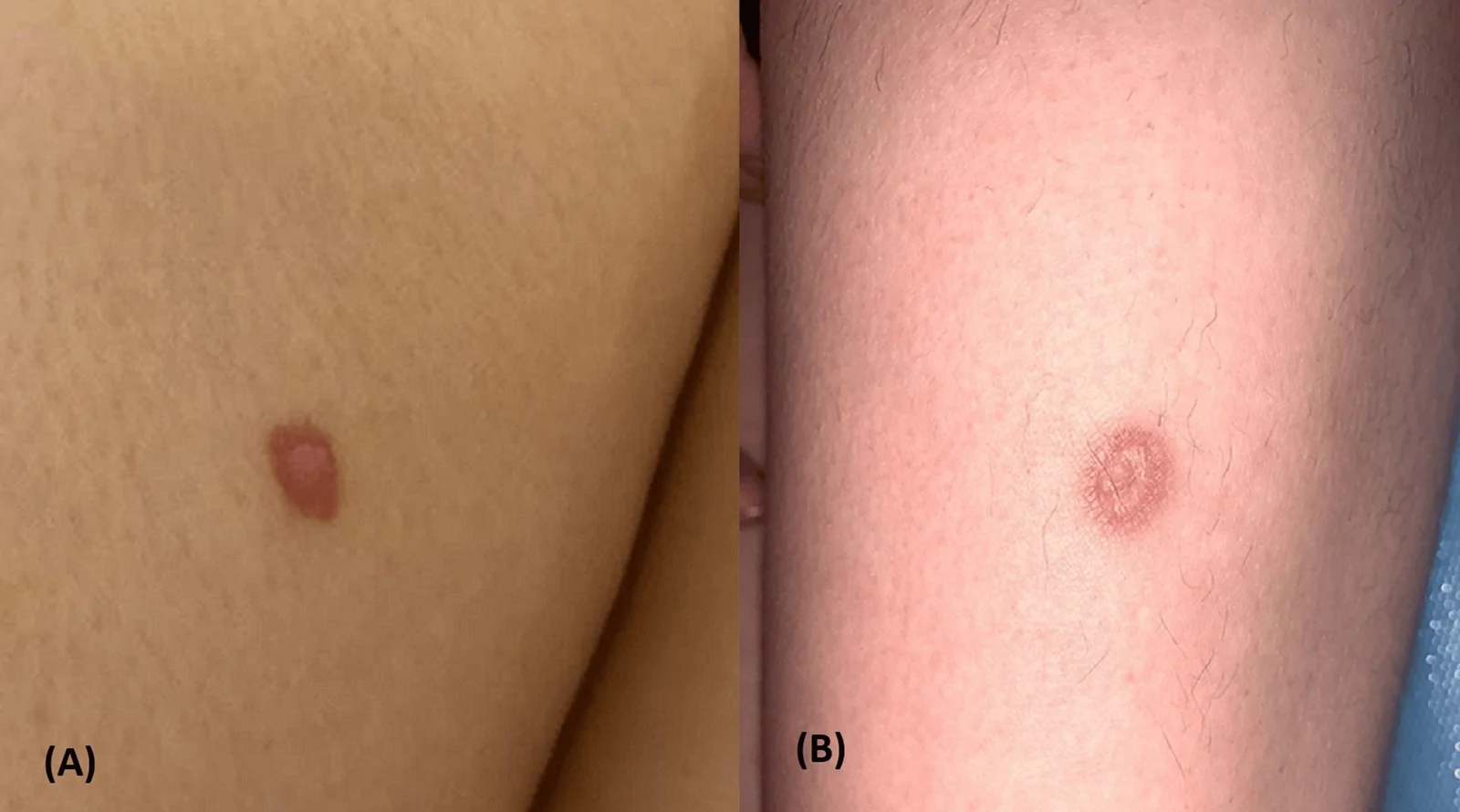
Clinical features Erythematous-pigmented plaque on the thigh (A: case 1, B: case 2).

**Figure 2:**
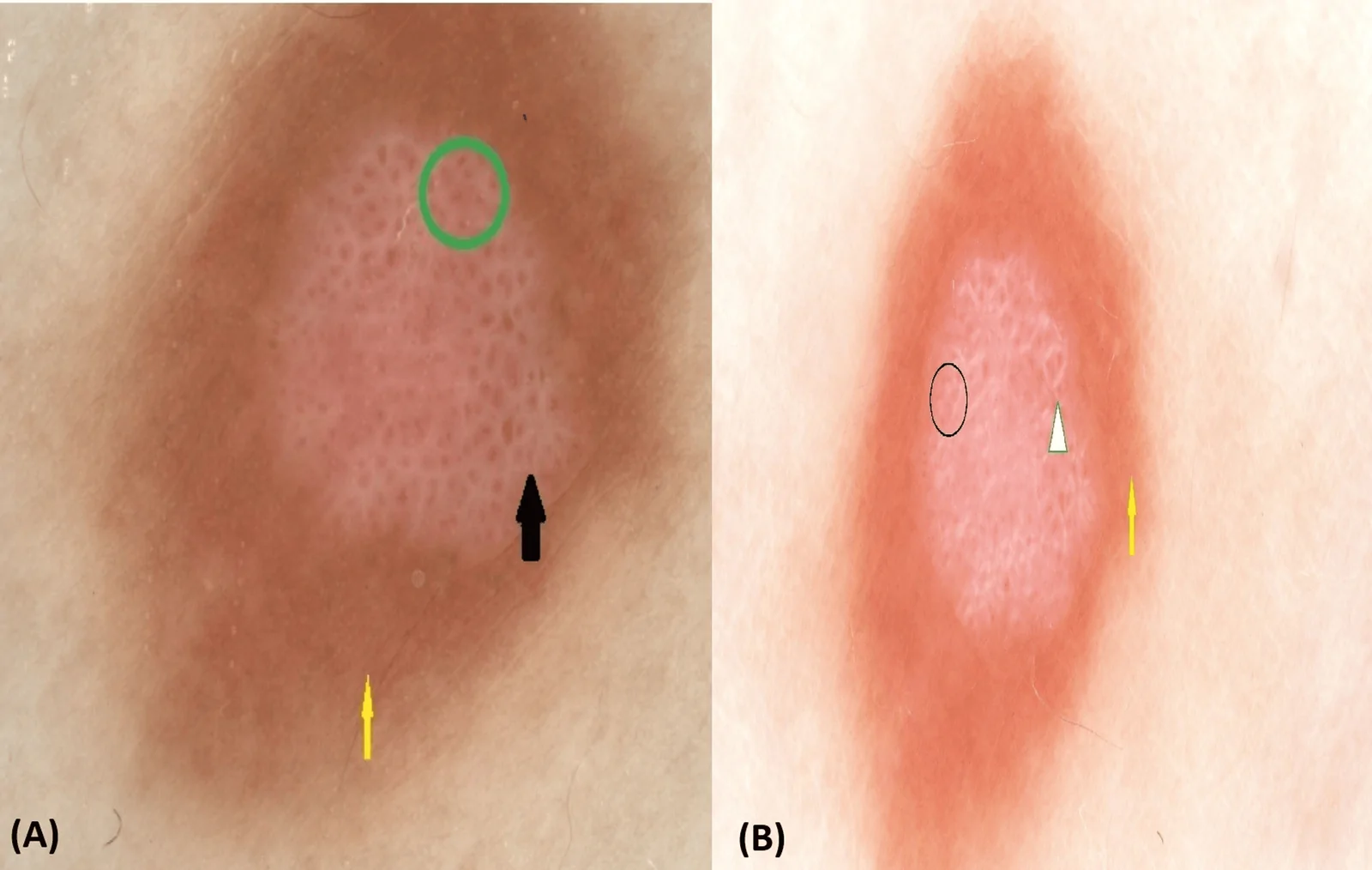
Dermoscopic features (case 1) (A) Non-polarized dermoscopy: inverse white network (black arrow), brown globules (green circle), and an atypical peripheral pigment network (yellow arrow). (B) Polarized dermoscopy: shiny white streaks (white triangle), dotted vessels (black circle), and a peripheral pigmented network (yellow arrow).

A 22-year-old woman with no relevant medical history presented with a four-month history of an erythematous-pigmented plaque on the thigh (Figure 1, Panel A). Dermoscopy revealed an asymmetric lesion with an inverse white network, brown globules, shiny white streaks (chrysalis), dotted vessels, and an atypical peripheral pigment network (Figure 2, Panels A and B). Although the clinical appearance initially suggested a dermatofibroma, the recent onset and multicomponent dermoscopic features raised suspicion of spitzoid melanocytic proliferation.

**Figure 3:**
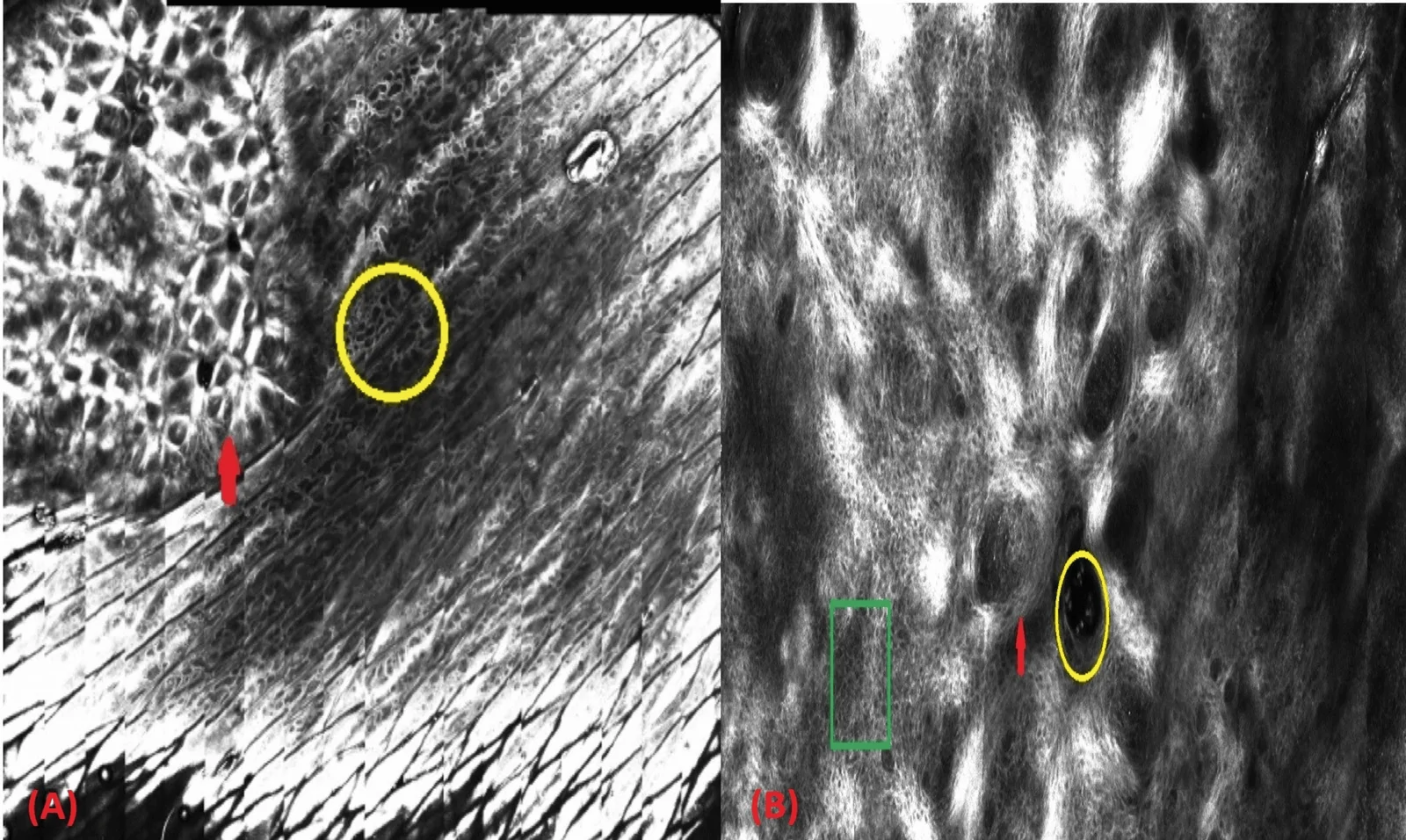
Reflectance confocal microscopy (case 1) (A) Highlighting a peripheral irregular ring pattern at the dermoepidermal junction (yellow circle) and bright collagen bundles (red arrow). (B) High magnification showing an irregular honeycomb pattern at the basal layer of the epidermis (green rectangle), poorly defined dermal papillae and scattered melanophages (yellow circle), and sparse dermal nests (red arrow).

RCM showed an overall asymmetric architecture and an irregular honeycomb pattern at the basal layer of the epidermis, corresponding to the inverse network observed on dermoscopy. Additional findings included bright collagen bundles correlating with the chrysalis structures seen dermoscopically, poorly defined dermal papillae, scattered melanophages, and sparse dermal nests. At the lesion periphery, an irregular ring pattern was observed at the dermoepidermal junction (Figure 3, Panels A and B).

**Figure 4:**
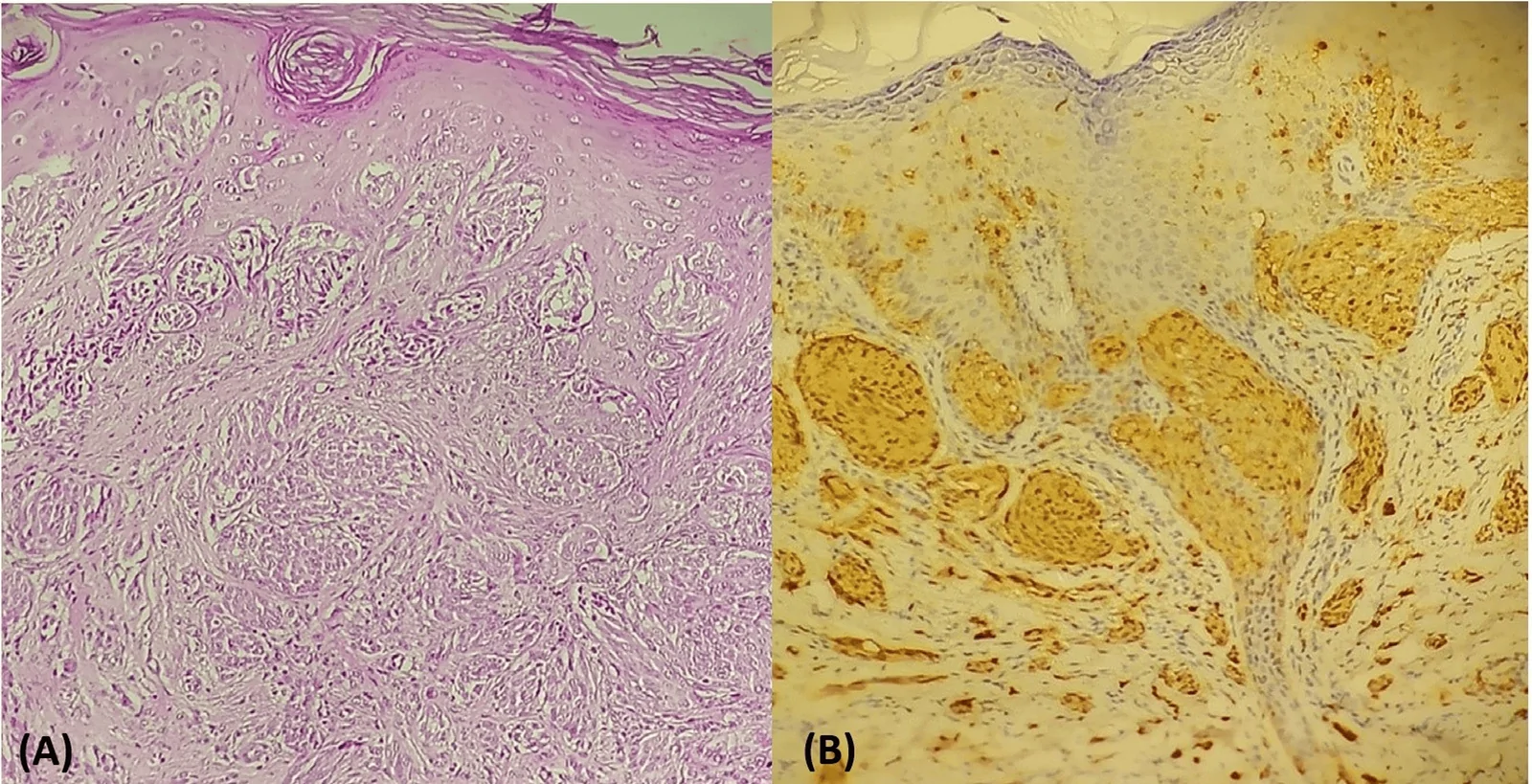
Immunohistopathology (case 1) (A) Histopathology showing a poorly circumscribed melanocytic proliferation with irregularly sized confluent nests of epithelioid and spindle-shaped spitzoid cells extending into the reticular dermis, without marked cytological atypia (H&E, ×100). (B) Immunohistochemistry showing diffuse and strong expression of S100.

Given these atypical findings, complete surgical excision was performed. Histopathological examination revealed a poorly circumscribed compound melanocytic proliferation involving both the junctional and dermal compartments and measuring more than 10 mm in greatest diameter. The lesion was composed of irregular and confluent nests of epithelioid and spindle-shaped spitzoid melanocytes with abundant pale cytoplasm and vesicular nuclei containing prominent nucleoli. Although no marked cytological atypia or deep dermal mitotic figures were identified, the architectural asymmetry, large confluent dermal nests, and extension into the reticular dermis supported the diagnosis of AST according to current histopathological criteria (Figure 4, Panel A). Immunohistochemical analysis demonstrated diffuse and strong expression of S100 (Figure 4, Panel B) and Melan-A, with heterogeneous p16 staining. Molecular analysis, which may provide additional diagnostic information in spitzoid lesions, could not be performed because these investigations were unavailable at our institution. At two years of follow-up, no local recurrence, regional lymph node involvement, or distant metastasis was observed.

Case 2

**Figure 5:**
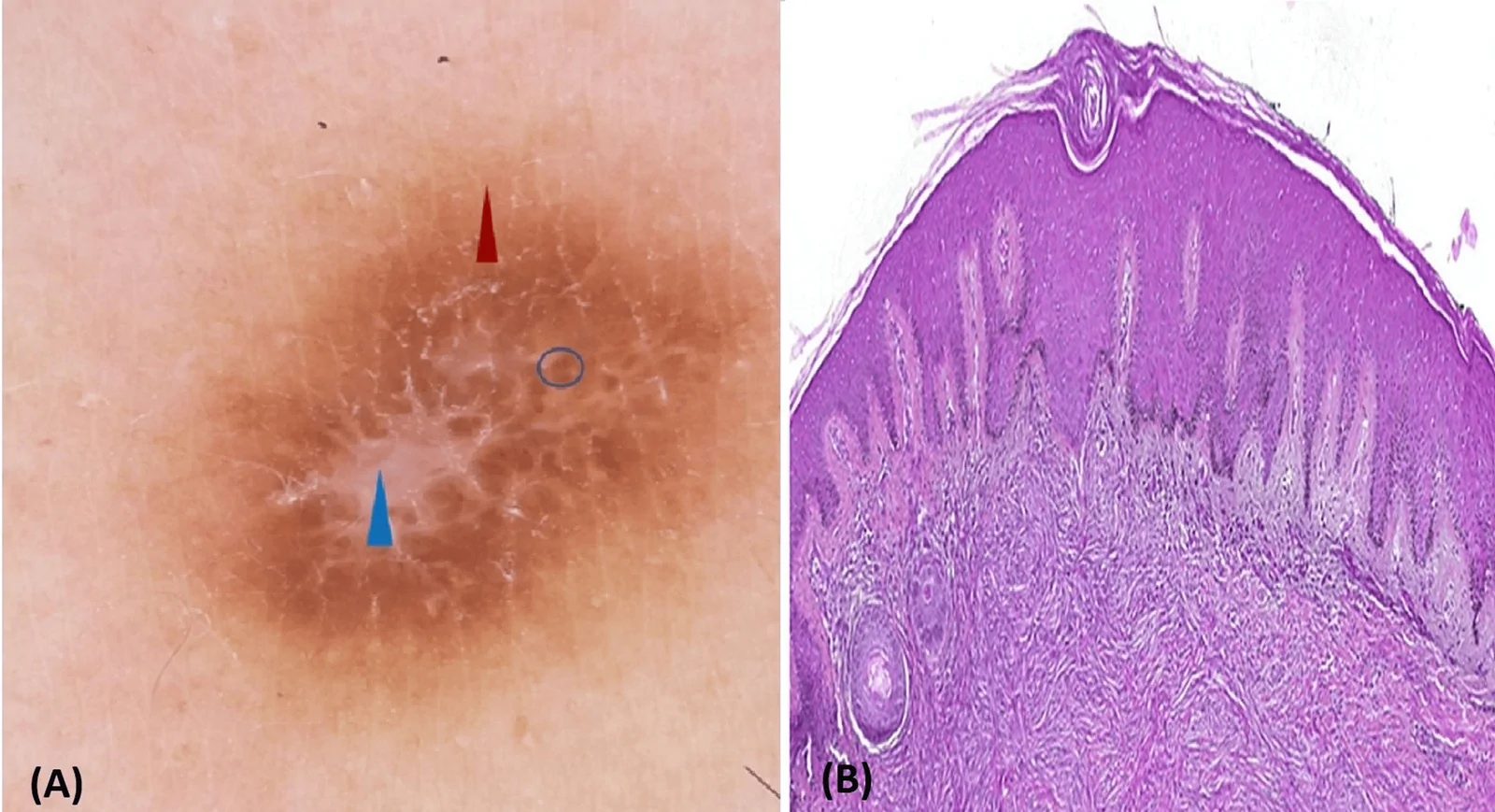
Dermatofibroma (case 2) (A) Dermoscopy showing a structureless white area (blue triangle), a typical peripheral pigmented network (red triangle), and brown ringed globules (blue circle). (B) Histopathology showing hyperplastic epidermis with hyperpigmented basal layer and a dermal proliferation of fibroblasts and spindle-shaped histiocytes without atypia (H&E, ×100).

A 25-year-old woman with no relevant medical history presented with a one-year history of an erythematous-pigmented plaque on the thigh (Figure 1, Panel B). Dermoscopy showed a structureless white area, a typical peripheral pigmented network, and brown ringed globules (Figure 5, Panel A), a classic pattern consistent with dermatofibroma. Excision was undertaken for aesthetic reasons and confirmed the diagnosis, demonstrating a hyperplastic epidermis with a hyperpigmented basal layer and a dermal proliferation of fibroblasts and spindle-shaped histiocytes without atypia (Figure 5, Panel B). No recurrence was observed after four years of follow-up. 

## Discussion

ASTs represent an intermediate category within the spitzoid spectrum between Spitz nevus and Spitz melanoma. Their diagnosis is fundamentally histopathological and requires an integrated assessment combining architectural, cytological, and, when available, molecular features rather than reliance on isolated criteria [1].

Clinically, the spitzoid lesions typically present as dome-shaped papules or nodules, pink to reddish-brown in color, measuring 5-10 mm or more, and predominantly located on the lower extremities of children and young adults [3]. Dermoscopic patterns vary according to pigmentation. Reported features include dotted vessels, chrysalis structures, inverse pigment network, and homogeneous pigmentation. Irregular pigment networks or peripheral globules may also be observed, reflecting significant overlap within the spitzoid spectrum and limiting the specificity of dermoscopy for definitive diagnosis [3,4].

In this context, the main diagnostic challenge is not the distinction between different entities within the spitzoid spectrum but rather the differentiation of spitzoid melanocytic proliferations from non-melanocytic mimickers such as dermatofibroma.

Dermatofibromas are benign fibrohistiocytic lesions presenting as firm papules, plaques, or nodules, often showing the dimple sign. Dermoscopically, they classically exhibit a peripheral delicate pigment network with a central white scar-like area. However, a wide spectrum of atypical dermoscopic patterns has also been described, which may occasionally pose diagnostic challenges [5].

Dermoscopy should be viewed as a diagnostic aid rather than a definitive tool in the evaluation of ASTs. By identifying suspicious spitzoid features and excluding alternative diagnoses, it helps guide the need for surgical excision and histopathological confirmation. Spitzoid lesions exhibiting asymmetric, multicomponent, or otherwise atypical dermoscopic patterns, particularly when ulcerated or rapidly growing, should be promptly excised [6].

RCM may also provide useful presurgical information by revealing junctional and dermal nests of hyperreflective melanocytic cells associated with architectural asymmetry. These features, while not pathognomonic, are compatible with a spitzoid melanocytic proliferation within the Spitz tumor spectrum. In this context, RCM contributes to strengthening the diagnostic suspicion [7].

From a histopathological standpoint, the diagnosis of an AST relies on consensus evaluation by experienced dermatopathologists. According to the WHO classification and current expert reviews, this diagnosis is based on an integrated assessment of architectural features (poor circumscription, confluence of nests, and extension into the reticular dermis), cytological features (epithelioid and spindle-cell morphology with variable nuclear pleomorphism), and mitotic activity, including possible deep mitoses, in correlation with clinical findings [1,6].

Molecular alterations, particularly kinase fusions involving *ALK*, *NTRK1/3*, *ROS1*, *RET*, or *BRAF*, may be identified and support the diagnosis in an appropriate morphological context. However, their absence does not exclude the diagnosis [6]. Molecular analysis was not performed due to unavailability at our institution.

Dermoscopic, RCM, and histopathological findings showed a close correlation in our case. The asymmetry observed on dermoscopy corresponded to architectural asymmetry on RCM and reflected the asymmetric melanocytic proliferation identified histologically. The inverse network seen dermoscopically correlated with an irregular honeycomb pattern at the dermoepidermal junction on RCM, corresponding histologically to epidermal acanthosis with elongated rete ridges. Brown globules observed on dermoscopy were associated with dermal nests on RCM and corresponded to melanocytic proliferation arranged in nests within both the epidermis and dermis on histopathological examination. In addition, shiny white streaks correlated with bright collagen bundles on RCM, consistent with stromal fibrosis and collagen remodeling. At the lesion periphery, the irregular pigmented network observed dermoscopically corresponded to an atypical ring pattern at the dermoepidermal junction on RCM and to irregular junctional melanocytic nests on histology. These clinicopathological correlations highlight the value of RCM as a noninvasive imaging technique that bridges dermoscopy and histopathology in the evaluation of atypical spitzoid lesions [7-9].

Regarding management, excision is generally recommended for spitzoid lesions in patients older than 12 years, given the age-associated increase in malignancy risk [10]. Complete surgical excision with clear margins remains the standard of care, as illustrated by the favorable two-year outcome observed in our patient.

## Conclusions

This report illustrates that spitzoid melanocytic proliferations and dermatofibromas may be clinically indistinguishable and that dermoscopy plays a key role in identifying features that should prompt histopathological investigation, even when the clinical impression is reassuring. In spitzoid lesions, the presence of dotted vessels, shiny white streaks, an inverse white network, and/or brown globules should raise suspicion of a melanocytic spitzoid proliferation. In contrast, the combination of a peripheral pigmented network with a central scar-like white area and/or brown ringed globules strongly supports the diagnosis of dermatofibroma. Despite advances in noninvasive imaging, including dermoscopy and RCM, the distinction between Spitz nevi, ASTs, and Spitz melanoma remains challenging. While these techniques provide important diagnostic clues, definitive classification relies on a multidisciplinary approach that integrates clinical, dermoscopic, histopathological, and increasingly molecular findings. The observations presented here should be considered illustrative insights from a case report, emphasizing the diagnostic challenges and practical value of recognizing key differentiating features among these entities.
